# Special Issue “African Swine Fever and Other Swine Viral Diseases in Africa”

**DOI:** 10.3390/v15071438

**Published:** 2023-06-26

**Authors:** Mary-Louise Penrith, Edward Okoth, Livio Heath

**Affiliations:** 1 Department of Veterinary Tropical Diseases, Faculty of Veterinary Science, University of Pretoria, Pretoria 0110, South Africa; 2 Biosciences, Animal and Human Health Program, International Livestock Research Institute (ILRI), Nairobi 00100, Kenya; 3 Transboundary Animal Diseases, Onderstepoort Veterinary Research, Agricultural Research Council, Pretoria 0110, South Africa

African swine fever (ASF) has become the swine disease of most global concern since its second escape from Africa in 2007 resulted in its spread to five continents and the consequent devastation of industrial to subsistence pig farming. This has triggered a great deal of research worldwide, as the disease manifests itself in different contexts. In sub-Saharan Africa, ASF is present in most countries where pigs are produced. The complexity of ASF is greatest in Africa, where the ASF virus originated and evolved in an ancient sylvatic cycle between a wild member of the pig family, namely the common warthog (*Phacochoerus africanus*), and soft ticks of the *Ornithodoros moubata* complex that live in the burrows and resting places occupied by the warthogs. This cycle is present in all eastern and southern African countries where it has been investigated. However, a marked increase in pig production in Africa in recent decades has been accompanied by an increase in ASF, and a cycle involving solely domestic pigs predominates in most sub-Saharan African countries, including West Africa, where the sylvatic cycle is absent. The increase in pig production has undoubtedly been accompanied by an increase in other viral diseases in swine, but the seven articles comprising this review, consisting of six original studies and one review, all relate to ASF.

In spite of various efforts spanning more than a century, there is still no safe and effective vaccine against ASF. Four of the seven studies described in this Special Issue are concerned with vaccine development. One of these is a general paper describing two novel epitopes on the CD2v protein of ASFV, a glycoprotein on the outer envelope of the virus that is of considerable interest in vaccine development [1]. The remaining three studies emanate from east Africa and are concerned with developing a vaccine to protect against infection with genotype IX ASFV, which was first described from a domestic pig isolate in Uganda, is dominant in East Africa and is a virus of concern due to increased propensity to spread in domestic pigs. Two studies investigated the safety and ability to protect against infection by vaccine candidates derived from deletions of target genes [2,3]. Although neither of the candidates were fully protective, the information is valuable to inform further initiatives to develop a safe and effective vaccine. The third study addresses a challenge for vaccine development by identifying a cell line for the propagation of a genotype IX isolate with retention of stability and virulence [4].

Although antigen detection is essential for early confirmation of ASF outbreaks because pigs usually die before antibodies reach detectible levels, antibody detection is useful for confirming earlier infection in a location with new incursions of the disease, in previous exposure to infection in endemic areas, as well as in vaccine development. A double antigen sandwich ELISA for the detection of antibodies to ASF that performed well in comparison to existing commercial kits, with advantages of low cost and short production time as described in the article, could form a useful addition to the existing diagnostic toolbox [5].

For more than half a century, outbreaks of ASF in domestic pigs in South Africa were restricted to an ASF-controlled area proclaimed in 1935 where the warthog–tick sylvatic cycle was endemic, with occasional spill over into the immediate environs of the area. However, extra-limital spread of warthogs has been widely reported, and the original study on *Ornithodoros* ticks from warthog burrows revealed ASD virus infection in ticks from several sites distant from the proclaimed endemic area [6].

The systematic review identified and categorized an impressive array of risk factors for African swine fever, both observed and potential, reported in the literature in various contexts over time and space [7]. This comprehensive literature search, for which the methodology was described in detail, provides a useful summary of a large proportion of the relevant literature related to risk factors for ASF.

We express our gratitude to all the authors for their contributions to this Special Issue. They provide encouragement by showing that research continues on aspects of ASF that are important for Africa, where the management of ASF has particular challenges which include a multiplicity of genotypes and a wildlife reservoir with an associated competent biological arthropod vector. This research provides pointers for the way forward in terms of further research, increased international collaboration and hope for progress in developing a safe and effective vaccine.
